# Ultra‐Thin SnO_x_ Buffer Layer Enables High‐Efficiency Quantum Junction Photovoltaics

**DOI:** 10.1002/advs.202204725

**Published:** 2022-10-26

**Authors:** Yuwen Jia, Haibin Wang, Yinglin Wang, Chao Wang, Xiaofei Li, Takaya Kubo, Yichun Liu, Xintong Zhang, Hiroshi Segawa

**Affiliations:** ^1^ Center for Advanced Optoelectronic Functional Materials Research and Key Laboratory of UV Light‐Emitting Materials and Technology of Ministry of Education Northeast Normal University Changchun Jilin 130024 P.R. China; ^2^ Graduate School of Arts and Sciences The University of Tokyo Tokyo 153–8902 Japan; ^3^ Research Center for Advanced Science and Technology The University of Tokyo Tokyo 153–8904 Japan

**Keywords:** capacitance effect, hysteresis, interfacial modification, quantum junction solar cells

## Abstract

Solution‐processed solar cells are promising for the cost‐effective, high‐throughput production of photovoltaic devices. Colloidal quantum dots (CQDs) are attractive candidate materials for efficient, solution‐processed solar cells, potentially realizing the broad‐spectrum light utilization and multi‐exciton generation effect for the future efficiency breakthrough of solar cells. The emerging quantum junction solar cells (QJSCs), constructed by n‐ and p‐type CQDs only, open novel avenue for all‐quantum‐dot photovoltaics with a simplified device configuration and convenient processing technology. However, the development of high‐efficiency QJSCs still faces the challenge of back carrier diffusion induced by the huge carrier density drop at the interface of CQDs and conductive glass substrate. Herein, an ultra‐thin atomic layer deposited tin oxide (SnO_x_) layer is employed to buffer this carrier density drop, significantly reducing the interfacial recombination and capacitance caused by the back carrier diffusion. The SnO_x_‐modified QJSC achieves a record‐high efficiency of 11.55% and a suppressed hysteresis factor of 0.04 in contrast with reference QJSC with an efficiency of 10.4% and hysteresis factor of 0.48. This work clarifies the critical effect of interfacial issues on the carrier recombination and hysteresis of QJSCs, and provides an effective pathway to design high‐performance all‐quantum‐dot devices.

## Graphical abstract

This work, for the first time introduces an ALD‐deposited ultrathin SnO_x_ buffer layer to further improve the performance of QJSCs. The authors in‐depth investigated the significant influence of interfacial carrier density difference on the *J*−*V* hysteresis of QJSCs and almost eliminated this hysteresis by the SnO_x_ buffer layer. This work paves an effective pathway to the development of high‐performance all‐quantum‐dot photovoltaic devices.

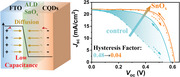

## 1 Introduction

**Figure 1:**
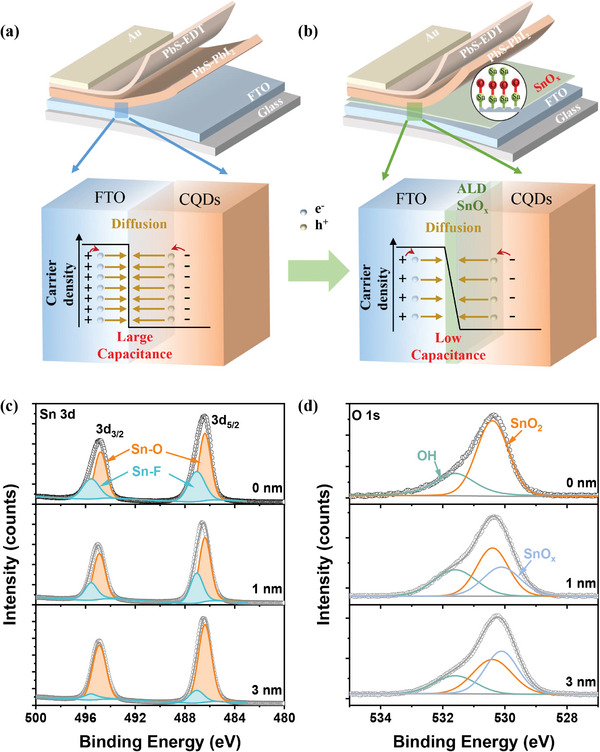
a,b) Schematic diagram of QJSCs with and without ALD SnO_x_ buffer layer. c) Sn 3d and d) O 1s XPS spectra of FTO covered by ALD SnO_x_ with different thickness.

Solution‐processed solar cells are promising for the cost‐effective development of photovoltaic technology.^[^ ^1^, ^2^, ^3^ ^]^ Lead sulfide (PbS) colloidal quantum dot solar cells (CQDSCs) have been adapted with various solution‐processed fabrication strategies,^[^ ^4^, ^5^, ^6^ ^]^ which are expected to achieve efficiency breakthrough due to the broad‐spectrum light utilization (300–1900 nm)^[^ ^7^, ^8^ ^]^ and multi‐exciton generation (MEG) effect.^[^ ^9^, ^10^ ^]^ Simplifying the materials and device structure of solar cells undoubtedly further improves the convenience and compatibility of the solution preparation method, and facilitates the fabrication of flexible,^[^ ^11^, ^12^ ^]^ tandem and integrated devices.^[^ ^13^, ^14^ ^]^ In particular, an emerging quantum junction device structure for efficient CQDSC, formed only by sequentially solution‐depositing n‐ and p‐type colloidal quantum dots (CQDs),^[^ ^15^, ^16^ ^]^ avoids other electron/hole extraction layers (EEL/HEL).^[^ ^17^, ^18^, ^19^ ^]^ This simplified quantum‐junction solar cell (QJSC) (**Figure** **1**) is therefore regarded as outstanding candidates for solution‐processed solar cells.^[^ ^20^ ^]^ In addition, QJSCs can easily realize the complementary absorption and band alignment matching by modulating the size and surface chemical of CQDs,^[^ ^15^ ^]^ and promote light‐soaking stability of devices by eliminating unstable metal oxide EEL^[^ ^21^ ^]^ and organic HEL.^[^ ^19^ ^]^ Recently, our work reported that QJSC has achieved a remarkable power conversion efficiency (PCE) of 10.5% through material and device optimization.^[^ ^20^ ^]^

Nevertheless, further efficiency improvement of QJSCs is still challenged by serious interfacial issues derived from the enormous carrier density difference between fluorine‐doped tin oxide (FTO) substrate and PbS CQD light‐active layer. FTO exhibits extremely high carrier density (≈10^20^—10^21^ cm^−3^) to guarantee its high conductivity.^[^ ^22^ ^]^ In contrast, the carrier density of PbS CQD is usually low (≈10^16^–10^17^ cm^−3^),^[^ ^23^, ^24^ ^]^ since the impurity doping of CQDs is a nontrivial assignment because of the dopant‐ion size mismatch and self‐purification during the crystal growth process.^[^ ^25^, ^26^ ^]^ As shown in Figure 1a, a tremendous carrier density drop at FTO/CQD interface causes the back carrier diffusion, and thus aggravates carrier recombination, especially the Auger recombination of CQD.^[^ ^27^ ^]^ It may block carrier collection,^[^ ^28^, ^29^ ^]^ causing the electron accumulation at the CQD layer and amplifying the interfacial capacitance.^[^ ^30^ ^]^ Previous literature have proved that the capacitance of solar cells is the main reason of current density−voltage (*J*−*V*) hysteresis behavior which causes an uncertain efficiency and a complicated test condition of solar cells.^[^ ^31^, ^32^, ^33^ ^]^ Similar back carrier diffusion phenomena caused by the carrier density drop was also reported in zinc oxide (ZnO)/CQDs heterojunction structure, and can be solved by inserting a thin insulting metal oxide or polymer block layer.^[^ ^34^, ^35^ ^]^ Therefore, solving the interfacial carrier density drops remains critical for high‐efficiency QJSCs.

Herein, we utilized an ultra‐thin tin oxide (SnO_x_) buffer layer, formed by atomic layer deposition (ALD) method, to solve the interfacial carrier density drop issues in QJSCs. ALD is an attractive method to prepare ultrathin, uniform, and pinhole‐free films with precise film thickness control. The ALD‐SnO_x_ layer with a low carrier density effectively buffered the carrier density variation at the FTO/PbS CQDs interface, thus attenuated the interfacial back carrier diffusion. Compared with the reference QJSC, ALD‐SnO_x_‐modified QJSCs showed significantly enlarged carrier lifetime and promoted carrier collection. Furthermore, the effect of ALD SnO_x_ buffer layer on transient capacitive currents of solar cells was systematically investigated, demonstrating the dramatical effect of ALD SnO_x_ buffer layer on eliminating *J*−*V* hysteresis of QJSCs. Finally, we successfully obtained a SnO_x_‐modified PbS QJSC with a record‐high PCE of 11.55% and an extreme low *J*−*V* hysteresis factor (HF) of 0.04, in contrast with the PCE of 10.40% and a HF of 0.48 for the reference QJSC.

## 2 Results and Discussion

The basic device structure of QJSCs (cell‐w/o) in our previous report^[^ ^20^ ^]^ is based on the quantum junction between n‐type PbI_2_‐capped PbS CQDs (PbS‐PbI_2_) and p‐type PbS CQDs capped with 1,2‐ethanedithiol (PbS‐EDT) as shown Figure 1a. In this work, we first prepared an ultra‐thin SnO_x_ buffer layer by ALD method on FTO substrate before the deposition of CQDs, fabricating a QJSC with a structure of FTO/SnO_x_/PbS‐PbI_2_/PbS‐EDT/Au (cell‐SnO_x_, Figure 1b). ALD strategy could realize a self‐limiting surface reaction and achieve a controlled monolayer deposition. Therefore, the thickness of ALD‐grown layers could be precisely controlled to several nanometers by altering the reaction cycles (Figure S1, Supporting Information). It was hardly observed this ALD SnO_x_ layer through scanning electron microscope (SEM, Figure S2, Supporting Information) because of its ultra‐thin thickness. However, the atomic force microscopy and SEM images of FTO covered by ALD SnO_x_ layer showed uniform surface topographies, suggesting the uniformity of this ALD SnO_x_ layer (Figure S3, Supporting Information). Ultraviolet‐visible (UV‐vis) absorption (Figure S4, Supporting Information), valence band‐X‐ray photoelectron spectroscopy (VB‐XPS, Figure S5, Supporting Information), and Kelvin probe force microscope (KPFM, Figure S6, Supporting Information) results also signified that the ultra‐thin SnO_x_ buffer layer had negligible effect on optical properties and band alignment at FTO/PbS interface. We further compared the XPS spectra of FTO covered by SnO_x_ layer with 0, 1, and 3 nm, respectively. As shown in Figure 1c, the peaks of Sn 3d_3/2_ and 3d_5/2_ at 495.59 and 487.09 eV were assigned to the Sn atom interacting with the doped F atoms in FTO, and those at 494.90 and 486.40 eV come from the oxidized Sn in FTO and ALD SnO_x_. The Sn‐F signals dropped dramatically along with the deposition cycles increasing, while the peak area ratio of Sn‐F to Sn‐O was decreased from 0.60 to 0.16, demonstrating the successful deposition of ALD SnO_x_ on the FTO substrate. In addition, O 1s spectra, as shown in Figure 1d, showed the appearance of amorphous SnO_x_ peak at 530.11 eV, further verifying the coverage of ALD SnO_x_ on FTO surface.

We explored the carrier density of the ALD SnO_x_ by the Mott–Schottky plots tested in Na_2_SO_3_ (0.5 M) electrolyte (Figure S7, Supporting Information), and the calculated carrier densities are listed in Table S1, Supporting Information. The FTO has a high carrier density of 8.51 × 10^20^ cm^−3^, which is consistent with the result of the previous literature obtained by the Hall effect.^[^ ^22^ ^]^ The 3‐nm‐thick SnO_x_ layer deposited on FTO exhibits a lower carrier density of 4.98 × 10^19^ cm^−3^. With the thickness of deposited SnO_x_ increasing, the carrier density of SnO_x_ decreases significantly. The 20‐nm‐thick SnO_x_ shows a carrier density of 2.53 × 10^18^ cm^−3^, which is similar to the reported carrier densities of ALD SnO_x_ obtained by Hall measurement.^[^ ^36^, ^37^ ^]^ The above results indicate that the electrons can diffuse from FTO substrate to SnO_x_ film. When the thickness of SnO_x_ layer is high (20 nm), the influence of FTO on the carrier density of SnO_x_ layer could be ignored. In contrast, 3 nm‐thick SnO_x_ can reduce the carrier density of the FTO/SnO_x_ surface by an order of magnitude, and can reduce the interfacial carrier density drop at the FTO/PbS‐PbI_2_ interface, as shown in Figure 1a.

### 2.1 Characterization of Device Performance

**Figure 2:**
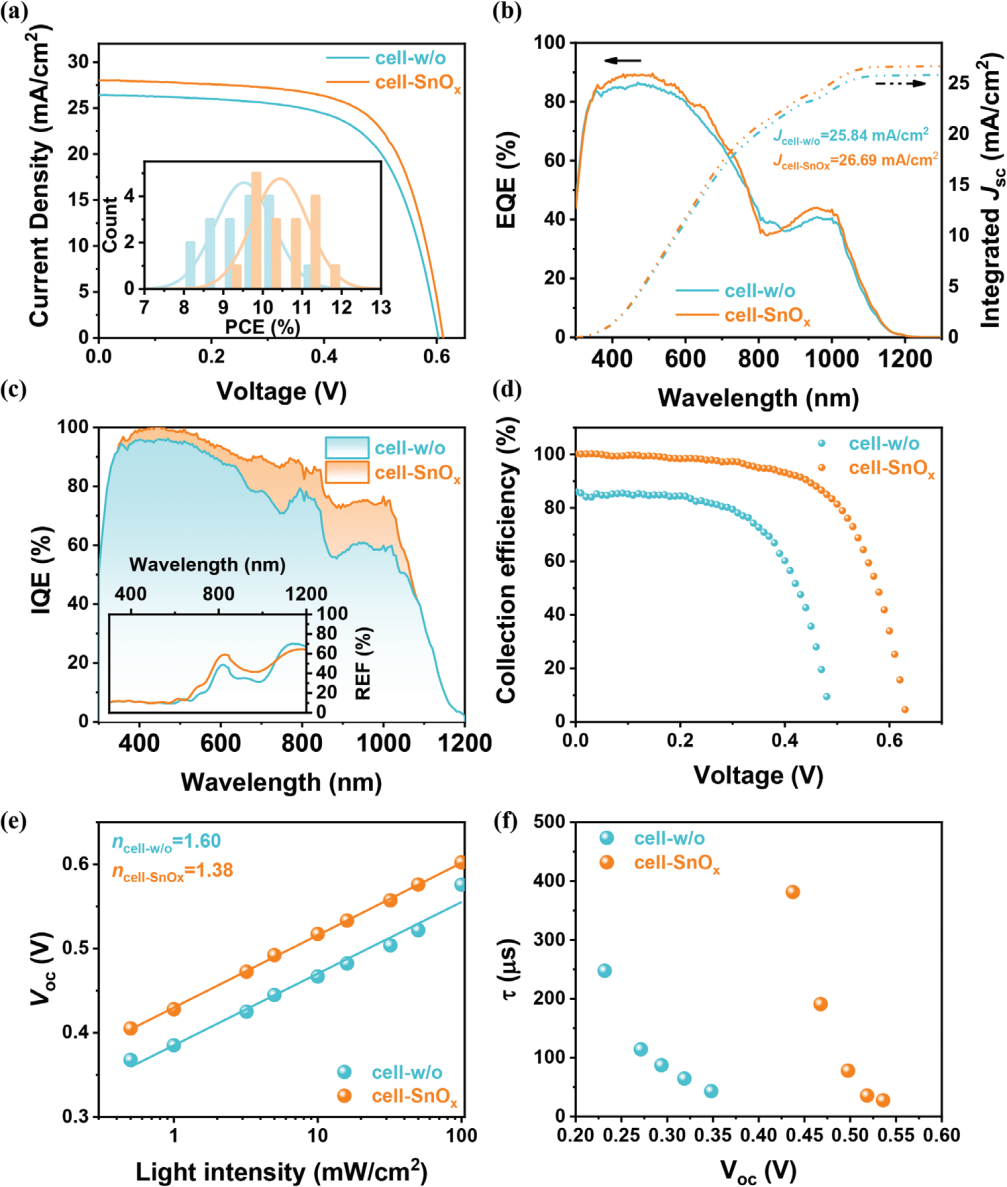
a) *J*−*V* characteristics of cell‐w/o and cell‐SnO_x_, which were tested under simulated illumination of 100 mW cm^−2^, AM 1.5G. The inset illustration was PCE statistical histogram calculated from 15 cell‐w/o and cell‐SnO_x_ devices. b) EQE and integrated *J*_sc_ curves of cell‐w/o and cell‐SnO_x_. c) IQE curves, reflectivity curves (inset), and d) carrier collection efficiency plotted against applied bias for cell‐w/o and cell‐SnO_x_. c) was the reflectivity of e) light‐intensity dependency of *V*_oc_ and f) carrier lifetime plots calculated from TPV measurement.

The thickness of ALD SnO_x_ buffer layer has been carefully optimized through comparing the photovoltaic performance of devices with 1, 3, 5, 10, and 20 nm SnO_x_ layer. A superior efficiency is derived from QJSCs with 3 nm SnO_x_ buffer layer (Figure S8, Supporting Information). To explore the effect of SnO_x_ buffer layer on the performance of QJSCs, we examined *J*−*V* curves of cell‐w/o and cell‐SnO_x_ under AM 1.5G, 100 mW cm^−2^ illumination. **Figure** **2**a and **Table** **1** showed that the cell‐w/o exhibited a short‐circuit current density (*J*_sc_) and an open‐circuit voltage (*V*_oc_) of 26.42 mA cm^−2^ and 0.603 V, respectively, while the cell‐SnO_x_ exhibited increased *J*_sc_ and *V*_oc_ of 28.02 mA cm^−2^ and 0.612 V, respectively. Consequently, the cell‐SnO_x_ achieved a PCE of 11.55% which is 11% higher than PCE of cell‐w/o (10.40%). We plotted the frequency distribution histogram of PCEs (Figure 2a) based on the efficiency parameters from 15 devices (Figure S9, Supporting Information and Table 1), proving that this ultra‐thin ALD SnO_x_ layer indeed improved the PCE of QJSCs. The improved *J*_sc_ of cell‐SnO_x_ was confirmed by the external quantum efficiency (EQE) test. The cell‐SnO_x_ exhibited a higher EQE thancell‐w/o in wavelength range from 400 to 1100 nm (Figure 2b). The corresponding integrated *J*_sc_ of cell‐w/o and cell‐SnO_x_ from EQE were 25.84 and 26.69 mA cm^−2^, respectively, which were consistent with the *J*−*V* results. Meanwhile, the unencapsulated cell‐SnO_x_ device showed considerable stability under both light‐soaking maximum power point tracking test and air‐storage condition (Figure S10, Supporting Information).

**Table 1:** Average and best‐performing *J*−*V* characteristic parameters of 15 cell‐w/o and cell‐SnO_x_ devices

	*J*_sc_[mA cm^−2^]	*V*_oc_[V]	FF[%]	PCE[%]	HF
cell‐w/o	25.35 ± 2.41 (26.42)	0.590 ± 0.015 (0.603)	63.47 ± 3.04 (65.30)	9.49 ± 1.02 (10.40)	0.51 ± 0.10 (0.48)
cell‐SnO_x_	26.82 ± 1.86 (28.02)	0.592 ± 0.014 (0.612)	65.78 ± 1.94 (67.40)	10.44 ± 0.71 (11.55)	0.11 ± 0.07 (0.04)

We further investigated the origin of this buffer‐layer‐induced performance enhancement by analyzing the internal quantum efficiency (IQE) and the carrier collection efficiency (CCE). The IQE can be calculated by Equation (1):

(1) $$IQE\;=\frac{EQE}{1-R}\;$$

where *R* is the reflectivity of solar cell. As shown in Figure 2c, IQE of cell‐SnO_x_ exhibited a significant improvement in a large wavelength range, which is higher than 95% in 355–560 nm and even achieved 100% in 430–470 nm. The photons with the wavelength of 430–470 nm possess energy larger than 2‐times *E*_g_ of the PbS QDs (1.33 eV) in our work, so the high quantum efficiency near 100% in this wavelength range is important for studying the MEG phenomena of CQDSCs in the further study.^[^ ^38^ ^]^ In addition, The CCE variation along with the applied voltage was calculated by the equation of

(2) $$\eta \;\left(V\right)=\frac{J_{1}\left(V\right)-J_{2}\left(V\right)}{J_{sc1}-J_{sc2}}\;\times \mathrm{IQE}\left(\lambda \right)$$

where *J*_1_(*V*) and *J*_2_(*V*) were current density measured under 635 nm laser illumination and dark, respectively; *J*_sc1_ and *J*_sc2_ were *J*_sc_ of solar cells under the aforementioned two irradiation condition; and IQE (*λ*) was the IQE value at 635 nm which were measured to be 86.85% and 90.55% for cell‐w/o and cell‐SnO_x_ from Figure 2c, respectively. As shown in Figure 2d, the cell‐SnO_x_ showed excellent CCE at different applied voltages, even achieving a remarkable value of 100% under bias at 0 V. In contrast, cell‐w/o generated an insufficient CCE of 86% at the same bias. As a result, the higher IQE and CCE are the main reasons of the ameliorated *J*_sc_ in cell‐SnO_x_.

The improvement of *V*_oc_ was also analyzed by serial electrical measurements. Firstly, we carried out Mott−Schottky analysis of cell‐w/o and cell‐SnO_x_. Both cells showed similar built‐in potential (*V*_bi_ = 0.58 V), indicating that the improved *V*_oc_ was not generated by the variation of *V*_bi_ (Figure S11, Supporting Information). We then investigated the effects of carrier recombination through the light‐intensity dependency of *V*_oc_ and *J*_sc_ (Figure S12, Supporting Information and Figure 2e), and estimated the recombination‐related ideal factor (*n*) of cell‐w/o and cell‐SnO_x_. (Equations S1 and S2, Supporting Information). The *n* of solar cells was reduced from 1.60 to 1.38 by ALD SnO_x_ modifying, suggesting that the trap‐related carrier recombination was suppressed in cell‐SnO_x_. We also obtained the carrier lifetime (*τ*) of cell‐w/o and cell‐SnO_x_ by transient photovoltage decay (TPV, Figure 2f) measurement. At the identical bias voltage, cell‐SnO_x_ presented longer *τ* than cell‐w/o, further proving the beneficial effect of ALD SnO_x_ layer on the suppression of carrier recombination in QJSCs. In addition, we tested steady‐state PL spectra of the FTO/PbS‐PbI_2_ and FTO/SnO_x_/PbS‐PbI_2_ samples to analyze the influence of ALD SnO_x_ on the carrier recombination of FTO/PbS‐PbI_2_ interface (Figure S13, Supporting Information). The FTO/PbS‐PbI_2_ shows a low emission peak at 1140 nm derived from PbS‐PbI_2_. After inserting the SnO*_x_* layer, the PL intensity of PbS‐PbI_2_ increases significantly. The integrated peak area of FTO/SnO_x_/PbS‐PbI_2_ is 4.57 × 10^8^, 7 times higher than that of FTO/PbS‐PbI_2_ sample (6.30 × 10^7^). This increased PL intensity also indicates that the SnO_x_ layer can reduce the nonradiative recombination at the FTO/PbS‐PbI_2_ interface.

### 2.2 *J* −*V* Hysteresis of QJSCs

**Figure 3:**
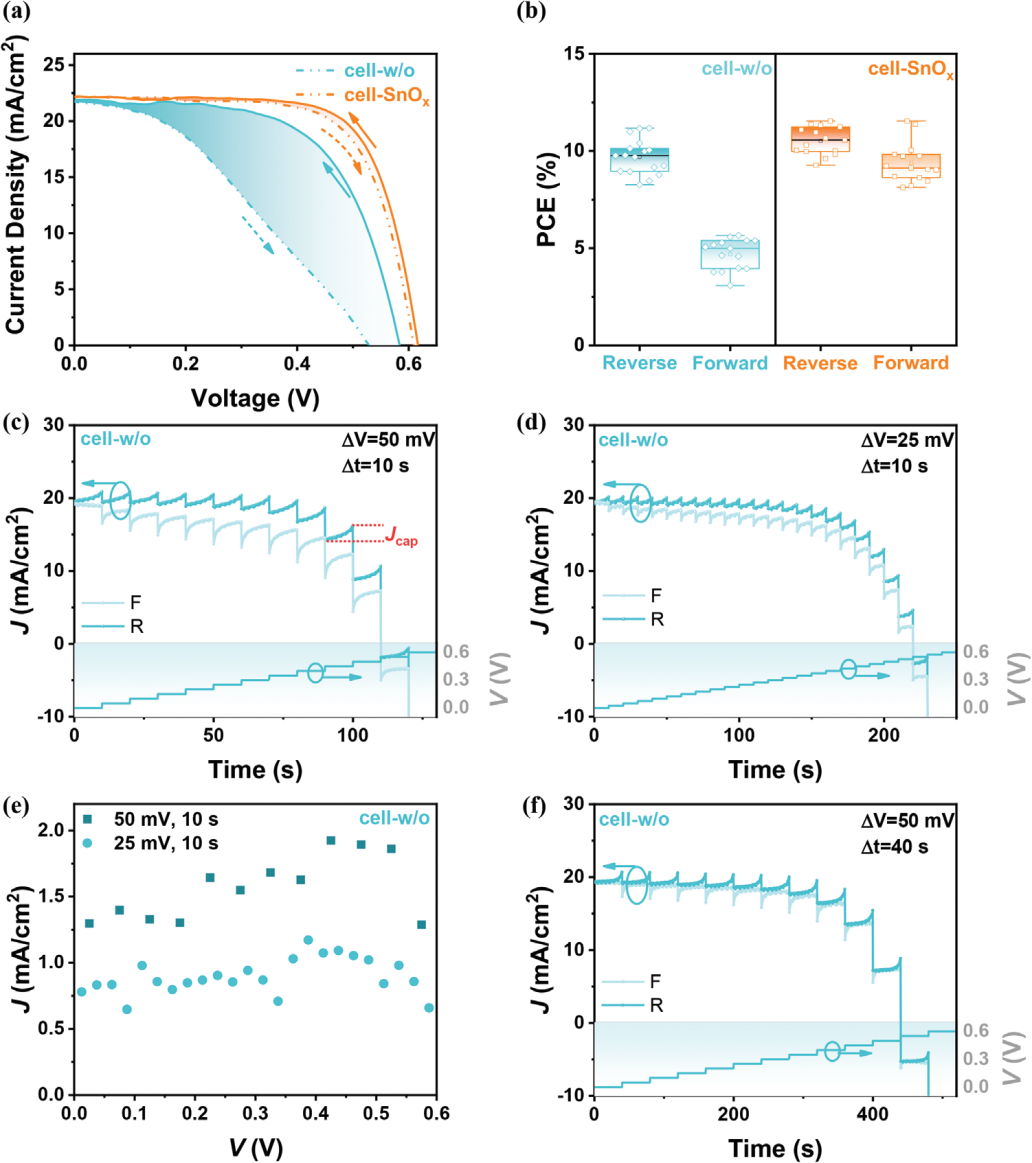
a) *J*−*V* curves of cell‐w/o and cell‐SnO_x_ in forward (dash) and reversed (solid) scan. b) PCEs of cell‐SnO_x_ in reverse and forward scan calculated from 15 cell‐w/o and cell‐SnO_x_ devices. Time‐dependent photocurrent response, tested under simulated illumination of 100 mW cm^−2^, AM 1.5G, under forward and reversed stepwise scan with c) 10 s step time and 50 mV step voltage and d) 10 s step time and 25 mV step voltage of cell‐w/o. e) The calculated *J*_cap_ variation of cell‐w/o in (c) and (d). f) The time‐dependent photocurrent response of cell‐w/o with 40 s step time and 50 mV step voltage.

It was notable that the ALD SnO_x_ buffer layer distinctly solved the *J*−*V* hysteresis problem of QJSCs. The *J*−*V* curves of cell‐w/o and cell‐SnO_x_ were characterized under forward (from 0 to 0.7 V) and reversed (from 0.7 to 0 V) scan, respectively, with a voltage step of 0.005 V and a scan rate of 0.05 V s^−1^. As shown in **Figure** **3**a, cell‐w/o exhibited noticeable *J*−*V* hysteresis behavior, indicated by the obvious PCE reduction during the froward scan. We calculated the hysteresis factor (HF) of cell‐w/o to be 0.48 from the equation of HF = (PCE_R_−PCE_F_)/PCE_R_, where PCE_R_ and PCE_F_ are PCE obtained from forward and reversed scan, respectively.^[^ ^39^, ^40^ ^]^ In contrast, cell‐SnO_x_ displayed ignorable *J*−*V* hysteresis and showed a small HF of 0.04 (Table 1). This effective effect of ALD SnO_x_ buffer layer on reducing *J*−*V* hysteresis of QJSCs was confirmed by 15 cells (Figure 3b).

**Figure 4:**
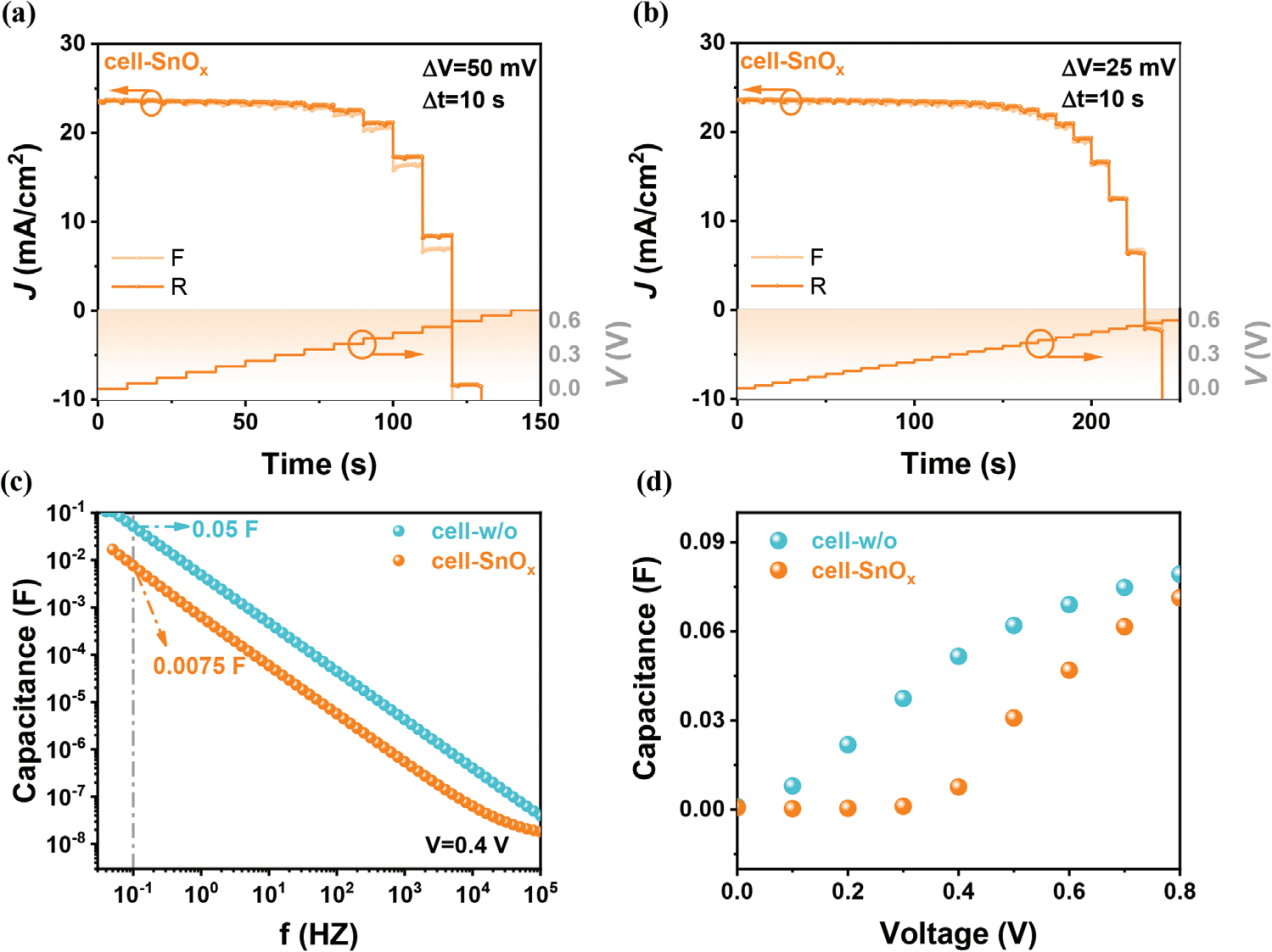
Time‐dependent photocurrent response of cell‐SnO_x_ measured with a) 10 s step time and 50 mV step voltage and b) 10 s step time and 25 mV step voltage. c) *C*−*f* plots measured under 0.4 V applied bias voltage. d) Capacitance changed with the applied bias voltage at the *f* of 0.1 Hz.

To deeply understand *J*−*V* hysteresis in these two cells, the time‐dependent photocurrent response (*J*−*t*) was investigated under stepwise forward and reverse scan (Figure 3c,d,f). The obtained current density of solar cells can be divided into steady‐state (*J*_ss_) and transient (*J*_t_) parts. The former one is the collected photocurrent density and reaches a steady‐state one at each end of voltage ramp step. The later one is equal to the variation of current density within each voltage step. We extracted the relation between *J*_t_ and applied bias from the *J*−*t* curves (Figure 3c,d), when the time step (Δ*t*) is 10 s and the voltage ramp steps (Δ*V*) are 50 and 25 mV, respectively (Figure 3e). These *J*_t_ of cell‐w/o slightly raise along with the increase of applied bias for both Δ*V* test condition, and achieved the maximum value around 0.4 V. In addition, *J*_t_ values of cell‐w/o at each applied bias with Δ*V* = 50 mV is about twice as much as those with Δ*V* = 25 mV. Based on this obtained dependence of *J*_t_ on the Δ*V*, we attributed this *J*_t_ of cell‐w/o to the capacitive current density (*J*_cap_). The *J*_cap_ is related to the capacitance according to equation of

(3) $$J_{\mathrm{cap}}=\frac{\Delta Q}{A\Delta t}\;=\frac{C\times \Delta V}{A\Delta t}\;$$

where *C* and *A* are the device capacitance and area, respectively.^[^ ^31^ ^]^ This formulation indicated that the *J*_cap_ of cell‐w/o should be doubled when Δ*V* increases from 25 to 50 mV, which was well constant with the dependence of *J*_t_ on the Δ*V* shown in Figure 3e. In contrast, the cell‐SnO_x_ showed ignorable *J*_t_ (*J*_cap_) in each test condition (**Figure** **4**a,b), suggesting that the SnO_x_ buffer layer effectively reduced the effect of capacitance on *J*−*V* hysteresis.

Capacitance−frequency (*C*−*f*) measurements displayed that cell‐SnO_x_ showed a lower capacitance value, compared with cell‐w/o (Figure 4c), at the same tested frequency. In addition, we verified *C*−*f* plots under different applied voltages (Figure S14, Supporting Information). Considering that the frequency of *C*−*f* tests should be associated with the reciprocal time step (Δ*t* = 10 s) in voltage scanning, we extracted capacitance variation along with the bias voltage at a low frequency of 0.1 Hz (Figure 4d). At the same bias voltage, the capacitance of cell‐SnO_x_ is much smaller than that of cell‐w/o (Figure 4d). For instance, the capacitance of cell‐w/o under the bias voltage of 0.4 V is 0.05 F cm^−2^ and generates a *J*_cap_ value of 2.37 mA cm^−2^ according to Equation (3), which indeed coincided with its *J*_t_ (*J*_cap_) in Figure 3e. While the capacitance of cell‐SnO_x_ under the same condition is only 0.35 mA cm^−2^, and thus induced an insignificant *J*_cap_ in the *J*−*t* test.

Furthermore, we increased the thickness of ALD SnO_x_ layer to 20 nm (Figure S8, Supporting Information), and the PCE of cell‐SnO_x_ was dramatically decreased. Considering that the effect of EEL in solar cell is to improve the carrier collection by the built‐in field in the space‐charge region, the reported thicknesses of EEL were usually larger than 10 nm (Table S2, Supporting Information) to make sure of formatting sufficient depletion width in the light‐harvesting layer. Based on the aforementioned optimized thickness of ALD SnO_x_ layer (3 nm) and the negligible influence of ALD SnO_x_ layer on the built‐in field obtained from capacitance−voltage (*C*−*V)* measurements (Figure S11, Supporting Information), we speculated that the ALD SnO_x_ layer in our work should not be an EEL, even though metal oxide, such as TiO_2_,^[^ ^41^ ^]^ ZnO^[^ ^42^ ^]^ and SnO_2_,^[^ ^17^, ^43^ ^]^ is generally used as EEL in solar cells. We further discuss the role of the ALD SnO_x_ layer in QJSCs. The PL spectrum has suggested that the SnO_x_ layer can reduce the carrier nonradiative recombination at the FTO/PbS‐PbI_2_ interface (Figure S13, Supporting Information). We attributed the non‐radiative carrier recombination of SnO_x_ sample to the buffering‐induced interface carrier density reduction. As shown in Figure 1a, in the cell‐w/o, the large difference in carrier density between FTO (≈10^20^ cm^−3^ obtained in *C*−*V* test) and PbS‐PbI_2_ (≈10^16^ cm^−3^) causes a drastic back diffusion of photo‐generated electrons from FTO and PbS‐PbI_2_ CQDs, which blocks carrier collection and aggravates the carrier recombination. Nevertheless, ALD SnO_x_ used in our work presents an intrinsic low carrier density (≈10^18^ cm^−3^, Table S1, Supporting Information). Carrier diffusion between FTO and ALD SnO_x_ ensures the gradual variation of carrier density at the FTO/SnO_x_/PbS interface, which effectively increases carrier collection efficiency and suppresses carrier recombination (Figure 1b).

*J*−*V* hysteresis have been observed in various types of solar cells.^[^ ^40^, ^44^ ^]^ Its origin is still critically debated and is proposed to ion migration,^[^ ^33^, ^45^ ^]^ charge trapping and detrapping,^[^ ^46^ ^]^ ferroelectric polarization,^[^ ^47^ ^]^ and capacitance effect.^[^ ^31^, ^32^ ^]^ Previous literature has reported the *J*−*V* hysteresis phenomena of PbS CQDSCs and attributed it to the bias‐stressed ionic migration, particularly protons from the surface ligands of CQDs.^[^ ^48^ ^]^ The effect of other factors on the hysteresis of PbS CQDSCs have not been discussed. Based on our *J*−*t* and capacitance analyses, it is reasonable to attribute the obvious *J*−*V* hysteresis of cell‐w/o to its large device capacitance. The large carrier density drop at FTO/PbS interface could cause a large interfacial capacitance, due to the interfacial carrier back diffusion. The charging and discharging of interfacial capacitance (Figure 1b) may generate the *J*−*V* hysteresis of QJSCs. As discussed above, the ALD SnO_x_ buffer layer is semiconductive, which could accept carrier diffused from FTO and buffer the carrier density drop at the FTO/PbS CQD interface, so cell‐SnO_x_ displayed insignificant capacitance‐induced *J*−*V* hysteresis behavior. To the best of our knowledge, this is the first report about the capacitance‐induced *J*−*V* hysteresis of CQDSCs. Our study demonstrated the important effect of the interfacial carrier density drops on CQDSCs hysteresis, and may attract more attention for the further understanding of *J*−*V* hysteresis mechanism in CQDSCs.

## 3 Conclusion

In summary, we utilized an ultra‐thin ALD SnO_x_ buffer layer to fabricate a high‐performance QJSCs with a record‐high efficiency of 11.55% and a low HF of 0.04. The optimized cell‐SnO_x_ significantly solved the critical issues of carrier recombination and carrier collection caused by the interfacial back carrier diffusion. Moreover, this SnO_x_ buffer layer effectively eliminated *J*−*V* hysteresis of solar cells, because it reduced the interfacial capacitance caused by the back carrier diffusion at FTO/PbS CQDs interface. Our work demonstrated the great potential of quantum junction device structure in developing high‐efficiency quantum dot photovoltaics, and provided a valuable scientific guide to deal with the interfacial recombination and capacitance problems for all‐quantum‐dot devices. QJSCs provides not only a promising bottom cell candidate for the tandem photovoltaics, but also a model device to investigate the physical mechanism of MEG‐induced photocurrent multiplication in the CQD‐based solar cells. Considering various advanced CQD materials possess tunable doping property, such as PbSe, perovskite QDs, and AgBiS_2_, we expect that more CQD systems could be introduced to construct QJSCs for further efficiency breakthrough.

## 4 Experimental Section

### Preparation of ALD SnO_x_ Film

The SnO_x_ buffer was prepared by the alternate deposition of Tetrakis (dimethylamino) tin (IV) (TDMASn, 99.9999%) as a tin source and deionized water as an oxidant, and the deposited thickness of each ALD cycle was ≈0.03 nm. Repeating the forward cycle, and getting the controlled thickness of SnO_x_ buffer layer.

### Synthesis of PbS‐OA CQD Solution

The synthesis of oleic‐acid‐capped PbS CQDs (PbS‐OA) was the same as in previously studies.^15^ The PbO (0.45 g) was dissolved in OA (2 ml) and ODE (18 ml), and the mixed solution (1) was degassed at 90 °C for 2 h. Then another mixed solution of 180 µl (TMS)_2_S and 2 ml OED was rapidly injected into the mixed solution (1) to synthesize PbS‐OA CQDs. The PbS‐OA CQD solution was then purified three times by acetone, and precipitated by centrifuge. Finally, the PbS‐OA CQDs was dissolved in octane with a concentration of 50 mg ml^−1^.

### Fabrication of the QJSCs

FTO was ultrasonically cleaned four times by detergent, ultra‐pure water, acetone, and ethanol, respectively. The SnO_x_ buffer layer was deposited on FTO by ALD. The PbS‐PbI_2_ solution, which was prepared by liquid‐phase ligand exchange showed in ,Supporting Information, was then spin‐coated on the FTO or FTO/SnO_x_ substrate at 2000 rpm for 30 s, followed by an annealing on 80 °C hotplate in the atmosphere. Then p‐type CQDs was filmed by layer‐by‐layer method. Finally, 100 nm Au was deposited as the electrode.

### Statistical Analysis

All results were derived from statistical results of at least 15 devices. The data in the table were presented as means±standard deviation (SD). Differences of data between the samples were analyzed using one‐way analysis of variance and student t‐test. A *p*‐value of <0.05 indicates that there is a significant difference between the groups of samples.

## Conflict of Interest

The authors declare no conflict of interest.

## Supporting information

Supporting Information

## Data Availability

The data that support the findings of this study are available from the corresponding author upon reasonable request.
