# Supplementary material for: Ultra‐Thin SnOx Buffer Layer Enables High‐Efficiency Quantum Junction Photovoltaics
Source: Adv Sci (Weinh). 2022 Oct 26;9(36):2204725. doi: 10.1002/advs.202204725 (PMC9799018; doi:10.1002/advs.202204725)
Supplement: Supplementary file 1 — Supporting Information [file ADVS-9-2204725-s001.pdf]

ADVANCED SCIENCE
Open Access

Supporting Information

for *Adv. Sci.*, DOI 10.1002/advs.202204725

Ultra-Thin SnO_x Buffer Layer Enables High-Efficiency Quantum Junction Photovoltaics

Yuwen Jia, Haibin Wang, Yinglin Wang, Chao Wang, Xiaofei Li, Takaya Kubo, Yichun Liu, Xintong Zhang* and Hiroshi Segawa*

Supplementary materials

Ultra-Thin SnO_x Buffer Layer Enables High-efficiency Quantum Junction Photovoltaics

Yuwen Jia, Haibin Wang, Yinglin Wang, Chao Wang, Xiaofei Li, Takaya Kubo, Yichun Liu, Xintong Zhang,* Hiroshi Segawa*

Y. Jia, Y. Wang, C. Wang, X. Li, Y. Liu, X. Zhang

Center for Advanced Optoelectronic Functional Materials Research, and Key Laboratory of UV Light-Emitting Materials and Technology of Ministry of Education, Northeast Normal University, Changchun 130024, Jilin. P.R. China

E-mail: xtzhang@nenu.edu.cn; wangyl100@nenu.edu.cn;

H. Wang, H. Segawa

Graduate School of Arts and Sciences, The University of Tokyo, 153-8902 Tokyo, Japan

T. Kubo, H. Segawa

Research Center for Advanced Science and Technology, The University of Tokyo, 153-8904 Tokyo, Japan

Keywords: quantum junction solar cells, hysteresis, capacitance effect, interfacial modification.

.

Supplementary material

Materials

Tetrakis (dimethylamino) tin (IV) (TDMASn, 99.9999%) were purchased from Suzhou Fornano Electronic Technology Co., Ltd. Ammonium acetate (99%) was purchased from Aladdin. Octane (99%+) was purchased from Acros. Acetonitrile (99.9%, extra dry with molecular sieves) was purchased from Innochem. Lead bromide ($PbBr_2$, for perovskite precursor, 99%), lead iodide (PbI_2, for perovskite precursor, 99.99%), N, N-Dimethylformanide (DMF, 99.5%, GC) were purchased from TCI. 1,2-Ethanedithiol (≥98.0%, GC) and butylamine (99.5%) were purchased from Sigma-Aldrich. Lead oxide (PbO, 99.9995%) was purchased from Alfa. Oleic Acid (OA, >90%), 1-Octadecene (ODE, 90%) and Hexamethyldisilathiane ($(TMS)_2S$, synthesis Grade) were purchased from Aldrich. Fluorine-doped tin oxide (FTO) glass with a sheet resistance of 8 ohm/square and a transmittance of >83% from Suzhou Shang Yang Solar Technology co., Ltd. was employed as transparent conductive substrate. All the chemicals were not reprocessed.

Liquid-phase ligand exchange process

The ligand-exchanged precursor solution was prepared by dissolved PbI_2 (0.1 M), $PbBr_2$ (0.05 M) and acetate ammonium (0.04 M) in 2.5 ml DMF. Then mixing 2.5 ml PbS-OA CQDs solution (octane, 20 mg/ml) with the precursor solution. After stirring vigorously, the PbS-PbI_2 CQDs was completely transferred from octane to DMF. The ligand-exchanged solution was washed 3 times by octane, and precipitated by toluene and centrifuge. After drying by vacuum, the PbS-PbI_2 solution was prepared by dissolving CQDs in butylamine with a concentration of 200 mg/ml.

Layer-by-layer process

50 mg/ml PbS-OA solution was spin-coated at 2500 rpm for 10 s. Then 1,2-ethanedithiol (EDT) acetonitrile solution (0.02 vol%) treated the film for 30 s, followed by acetonitrile washing 3 times.

Characterization methods

X-ray photoelectron spectroscopy (XPS) was carried out by X-ray photoelectron

spectrometer (Thermo Scientific K-Alpha system) with an Al Kα source.

UV-visible near infrared absorption spectrum was measured by HITACHI U4150. The scanning speed was 600 nm/min, and the scanning step was 1 nm.

Scanning electron microscope (SEM) images were captured through Sigma 300 (ZEISS).

Atomic force microscope (AFM) images were obtained through Bruker Dimension Icon instrument (Bruker, Berlin, Germany). The surface topography was measured using Silicon Tip on Nitride Lever (model: scanasyst-air), and the Kelvin probe force microscope was measured by the 0.01-0.025 Ohm-cm Antimony (n) doped Si (model: SCM-PIT-V2).

Current density–voltage (*J*–*V*) characteristic was measured by Keithley 2632 source meter under simulated 100 mW cm^{-2}, AM1.5G illumination (SS-F5-3A, Enlitech)

External quantum efficiency (EQE) and reflectivity (*R*) were performed by Solar Cell Spectral Responsivity, Reflectance and Quantum Efficiency System (Zolix Instruments Co., Ltd.). The test step of EQE and reflectivity were 5 nm.

Light intensity dependency of J_{sc} and V_{oc}. We can analysis the exponential factor (α) from J_{sc} vs light intensity (P) test, using the relational expression of

$$J_{sc} \propto P^{\alpha} \quad \text{(S1)}$$

The ideal factor (n) and reverse saturation current density (J_0) can be expressed by

$$V_{oc} = \frac{nkT}{q}\ln(\frac{J_{sc}}{J_0}) \quad \text{(S2)}$$

where k is Boltzmann constant, T is temperature, and q is elementary charge.

Mott-Schottky measurement, transient photovoltage decay (TPV) and capacitance–frequency (*C*-*f*) were measured by Modulab XM PhotoEchem station (Solartron Analytical). The frequency of Mott-Schottky measurement was 1000 Hz, and the step of voltage was 5 mV.

The Mott-Schottky test of the solution method was carried out by the Advanced Electrochemical System PARSTAT 2273 (Princeton Applied Research) in the Na_2SO_3 electrolyte (0.5 M) at 1000 Hz. The tested voltage range was -500-600 mV, and the sweep step was 20 mV/s. The carrier density (n) could be calculated from the slope of

the linear region by the equation:

$$\frac{d\frac{1}{C^2}}{dV} = \frac{2}{A^2 q \varepsilon \varepsilon_0 n} \tag{S3}$$

where ε_0 is vacuum permittivity, ε is the dielectric constant of SnO_x, which in the used as 10 according the reported literature [1], q is the elementary charge and A is the area of tested sample (1.2 cm^2).

Carrier collection efficiency was measured by Keithley 2632 source meter with class IV laser product (MRL-III-635-500 mW, Changchun New Industries optoelectronics tech Co., Ltd. (CNI)) illumination under a current of 0.35 A.

Photoluminescence (PL) spectroscopy was tested by the QuantaMaster™ 8000 (Horiba Scientific) with a near-infrared detector (H10330C-75-C2), and a 635 nm laser was used as the excitation light.

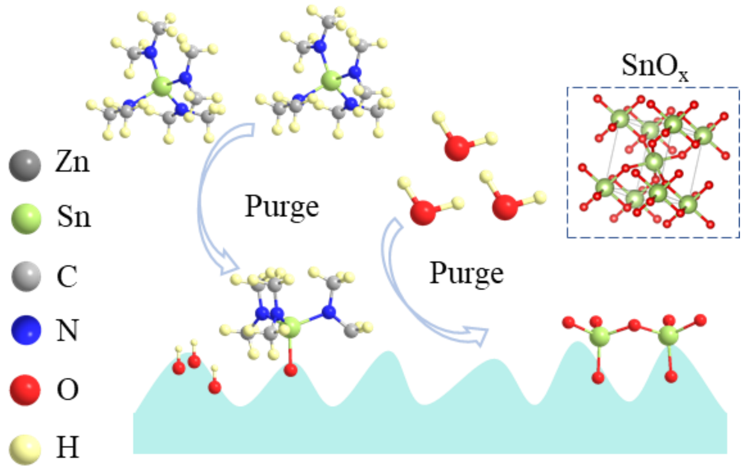

Figure S1. Schematic diagram of the SnO_x buffer layer growth process deposited by atomic layer deposition (ALD) method.

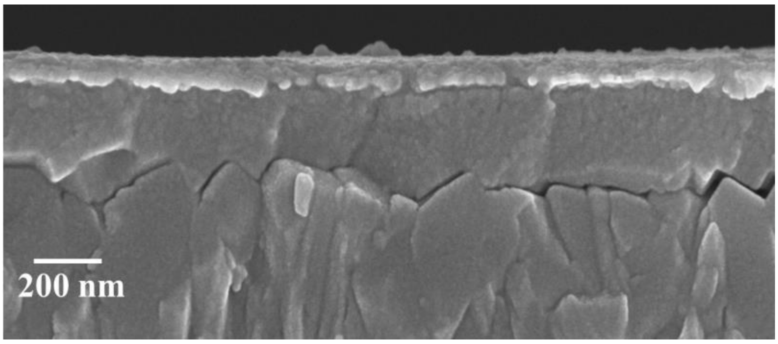

Figure S2. Cross-sectional scanning electron microscope (SEM) image of cell-SnO_x.

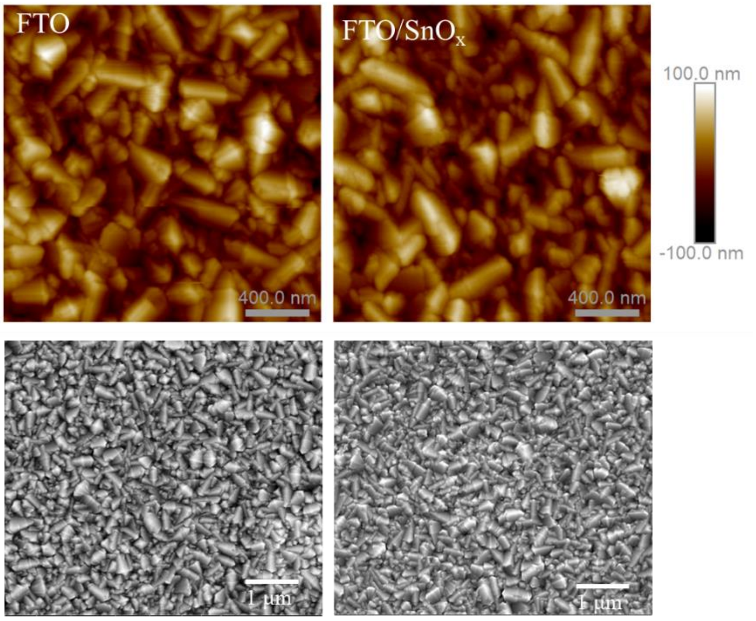

Figure S3. Surface topography of FTO and FTO/SnO_x samples, which were measured by atomic force microscope (AFM) and scanning electron microscope (SEM).

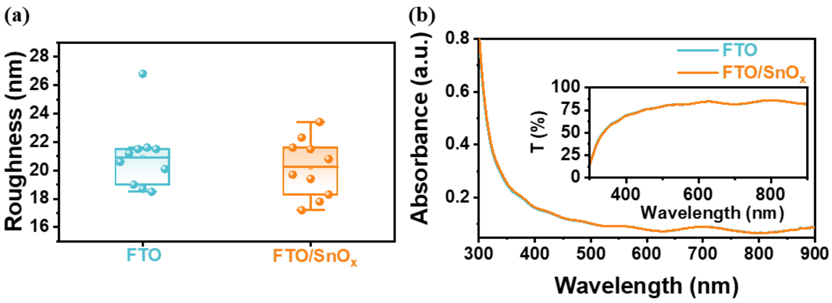

Figure S4. (a)Roughness of FTO and FTO/SnO_x samples calculated from AFM. (b) Absorbance and transmittance spectrum of FTO and FTO/SnO_x.

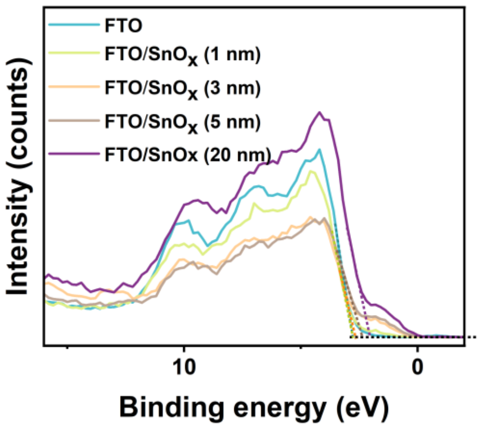

Figure S5. VB-XPS spectrum of the FTO substrate and FTO with SnO_x in different thickness (1, 3, 5, 20 nm), and the corresponding E_F-VB offsets were 2.79, 2.78, 2.73,

2.36 and 2.04 eV.

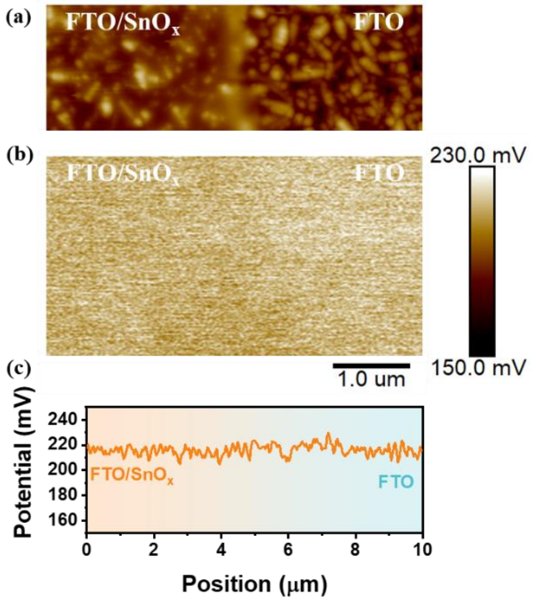

Figure S6. (a) Surface topography of FTO, in which the left side was FTO with 3-nm-thikc SnO_x buffer layer, and the right side was bare FTO substrate. Corresponding (b) surface potential images of KPFM and (c) Cross section data of KPFM.

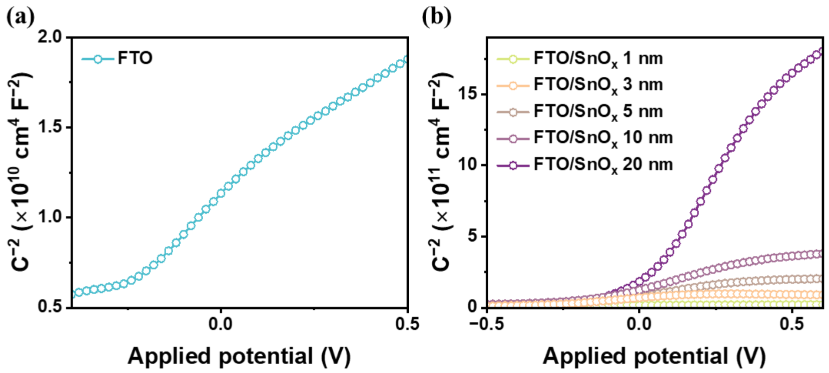

Figure S7. (a) The Mott-Schottky plots of the FTO substrate and (b) FTO/SnO_x sample with different thickness of ALD SnO_x, which were tested by the Advanced Electrochemical System (Parstat 2273) in Na_2SO_3 (0.5 M) electrolyte at 1000 Hz.

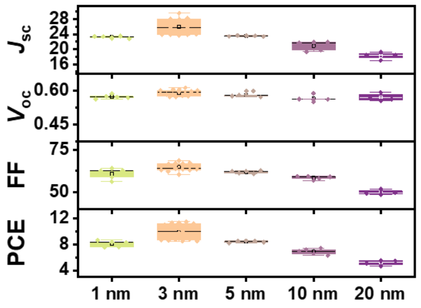

Figure S8. Average performance parameters of 15 devices calculated from *J*–*V* curves

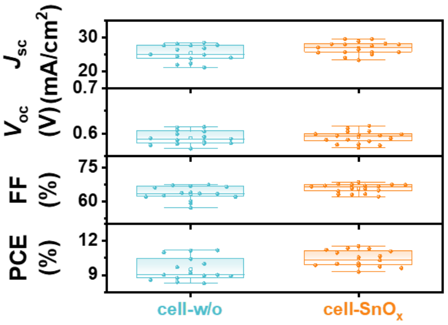

Figure S9. Average performance parameters of 15 cell-w/o and cell-SnO$_x$ devices calculated from *J*–*V* curves.

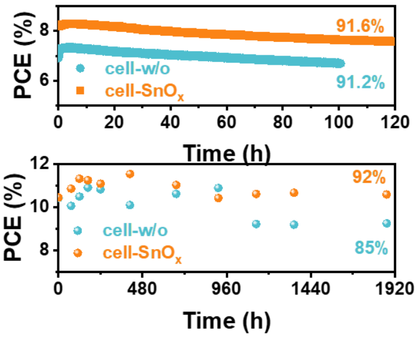

Figure S10. Monitored stability of cell-w/o and cell-SnO$_x$, including MPPT plots (top side) and PCE evolution with the air-storage time (bottom side)

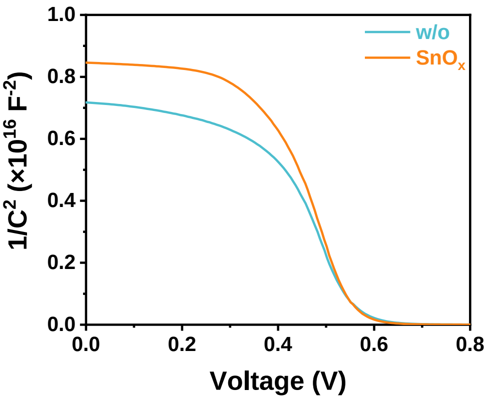

Figure S11. Mott-Schottky plot of FTO /PbS-PbI_2/Au (w/o) and FTO/SnO_x/PbS-PbI_2/Au (SnO_x) samples.

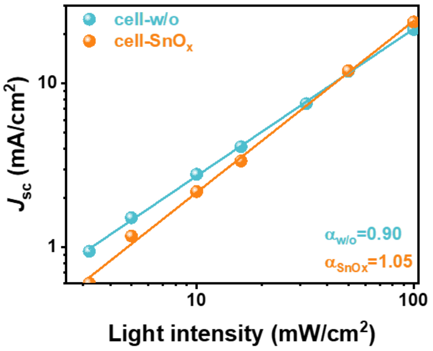

Figure S12. light intensity dependency of J_{sc} of cell-w/o and cell-SnO_x

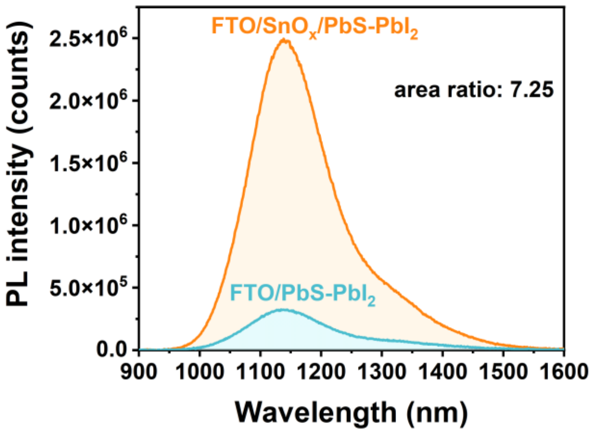

Figure S13. Steady-state PL spectrum of FTO/PbS-PbI_2 and FTO/SnO_x/PbS-PbI_2 films, which are tested under the excitation of 635 nm laser.

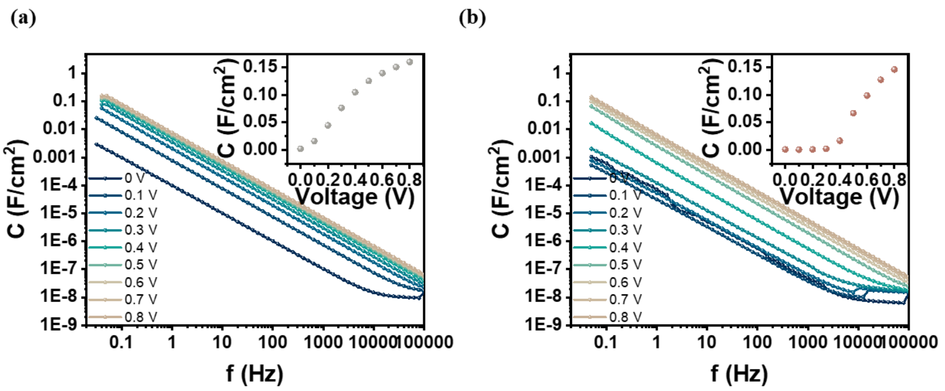

Figure S14. *C*−*f* plots of (a) cell-w/o and (b) cell-SnO_x devices under different applied voltages.

Table S1. The carrier density of the FTO and ALD SnO_x, which are calculated from the Mott-Schottky measurement.

Sample	FTO	FTO/SnO_x (1 nm)	FTO/SnO_x (3 nm)	FTO/SnO_x (5 nm)	FTO/SnO_x (10 nm)	FTO/SnO_x (20 nm)
Carrier density (cm^{-3})	8.51×10^{20}	3.61×10^{20}	4.98×10^{19}	2.68×10^{19}	1.57×10^{19}	2.53×10^{18}

Table S2. Previous works of the ALD-SnO_x in perovskites solar cells and SnO_2 in colloidal quantum dot solar cells.

Ref	Field	Method	Thickness	Function
RSC Adv., 2015, 5, 28424. [2]	PSCs	spin-coating	200 nm	ETL
Adv. Mater. 2017, 29, 1703852.[3]	PSCs	spin-coating	25 nm	ETL
Energy Environ. Sci., 2015, 8, 2928.[4]	PSCs	ALD	15 nm	ETL
Energy Environ. Sci., 2016, 9, 81.[5]	perovskite/silicon tandem solar cells	ALD	15 nm	ETL
J. Mater. Chem. A, 2016, 4, 12080.[6]	PSCs	ALD	15 nm	ETL
ACS Appl. Mater. Interfaces 2017, 9, 29707.[7]	PSCs	ALD	53 nm	ETL

ACS Energy Lett. 2017, 2, 2118.[8]	Flexible PSCs	ALD	60 nm	ETL
Adv. Energy Mater. 2017, 7, 1700414.[1]	PSCs	ALD	40 nm	ETL
Adv. Sci. 2018, 5, 1800130.[9]	PSCs	ALD	20 nm	ETL
Chem. Commun., 2019, 55, 2433.[10]	PSCs	ALD	12 nm	ETL
Energy Environ. Sci., 2017, 10, 1207.[11]	PSCs	ALD	15 nm	ETL
Nat. Energy, 2017, 2, 17009.[12]	perovskite/silicon tandem solar cells	ALD	4 nm	Window layer/buffer layer
Adv. Energy Mater. 2018, 8, 1800591.[13]	PSCs	ALD	6 nm	Buffer layer
J. Mater. Chem. A, 2017, 5, 17240.[14]	CQDSCs	spin-coating	100 nm	ETL

References

[1] Wang, C.; Xiao, C.; Yu, Y.; Zhao, D.; Awni, R. A.; Grice, C. R.; Ghimire, K.; Constantinou, I.; Liao, W.; Cimaroli, A. J.; Liu, P.; Chen, J.; Podraza, N. J.; Jiang, C. S.; Al-Jassim, M. M.; Zhao, X.; Yan, Y., *Adv. Energy Mater.* **2017,** *7* (17), 1700414.

[2] Li, Y.; Zhu, J.; Huang, Y.; Liu, F.; Lv, M.; Chen, S.; Hu, L.; Tang, J.; Yao, J.; Dai, S., *RSC Advances* **2015,** *5* (36), 28424-28429.

[3] Jiang, Q.; Chu, Z.; Wang, P.; Yang, X.; Liu, H.; Wang, Y.; Yin, Z.; Wu, J.; Zhang, X.; You, J., *Adv. Mater.* **2017,** *29* (46), 1703852.

[4] Correa Baena, J. P.; Steier, L.; Tress, W.; Saliba, M.; Neutzner, S.; Matsui, T.; Giordano, F.; Jacobsson, T. J.; Srimath Kandada, A. R.; Zakeeruddin, S. M.; Petrozza, A.; Abate, A.; Nazeeruddin, M. K.; Grätzel, M.; Hagfeldt, A., *Energy Environ. Sci.* **2015,** *8* (10), 2928-2934.

[5] Albrecht, S.; Saliba, M.; Correa Baena, J. P.; Lang, F.; Kegelmann, L.; Mews, M.;

Steier, L.; Abate, A.; Rappich, J.; Korte, L.; Schlatmann, R.; Nazeeruddin, M. K.; Hagfeldt, A.; Grätzel, M.; Rech, B., *Energy Environ. Sci.* **2016,** *9* (1), 81-88.

[6] Wang, C.; Zhao, D.; Grice, C. R.; Liao, W.; Yu, Y.; Cimaroli, A.; Shrestha, N.; Roland, P. J.; Chen, J.; Yu, Z.; Liu, P.; Cheng, N.; Ellingson, R. J.; Zhao, X.; Yan, Y., *J. Mater. Chem. A* **2016,** *4* (31), 12080-12087.

[7] Hultqvist, A.; Aitola, K.; Sveinbjornsson, K.; Saki, Z.; Larsson, F.; Torndahl, T.; Johansson, E.; Boschloo, G.; Edoff, M., *ACS Appl. Mater. Interfaces* **2017,** *9* (35), 29707-29716.

[8] Wang, C.; Guan, L.; Zhao, D.; Yu, Y.; Grice, C. R.; Song, Z.; Awni, R. A.; Chen, J.; Wang, J.; Zhao, X.; Yan, Y., *ACS Energy Lett.* **2017,** *2* (9), 2118-2124.

[9] Lee, Y.; Lee, S.; Seo, G.; Paek, S.; Cho, K. T.; Huckaba, A. J.; Calizzi, M.; Choi, D. W.; Park, J. S.; Lee, D.; Lee, H. J.; Asiri, A. M.; Nazeeruddin, M. K., *Adv. Sci.* **2018,** *5* (6), 1800130.

[10] Jeong, S.; Seo, S.; Park, H.; Shin, H., *Chem. Commun.* **2019,** *55* (17), 2433-2436.

[11] Correa-Baena, J.-P.; Tress, W.; Domanski, K.; Anaraki, E. H.; Turren-Cruz, S.-H.; Roose, B.; Boix, P. P.; Grätzel, M.; Saliba, M.; Abate, A.; Hagfeldt, A., *Energy Environ. Sci.* **2017,** *10* (5), 1207-1212.

[12] Bush, K. A.; Palmstrom, A. F.; Yu, Z. J.; Boccard, M.; Cheacharoen, R.; Mailoa, J. P.; McMeekin, D. P.; Hoye, R. L. Z.; Bailie, C. D.; Leijtens, T.; Peters, I. M.; Minichetti, M. C.; Rolston, N.; Prasanna, R.; Sofia, S.; Harwood, D.; Ma, W.; Moghadam, F.; Snaith, H. J.; Buonassisi, T.; Holman, Z. C.; Bent, S. F.; McGehee, M. D., *Nature Energy* **2017,** *2* (4).

[13] Palmstrom, A. F.; Raiford, J. A.; Prasanna, R.; Bush, K. A.; Sponseller, M.; Cheacharoen, R.; Minichetti, M. C.; Bergsman, D. S.; Leijtens, T.; Wang, H. P.; Bulović, V.; McGehee, M. D.; Bent, S. F., *Adv. Energy Mater.* **2018,** *8* (23).

[14] Khan, J.; Yang, X.; Qiao, K.; Deng, H.; Zhang, J.; Liu, Z.; Ahmad, W.; Zhang, J.; Li, D.; Liu, H.; Song, H.; Cheng, C.; Tang, J., *J. Mater. Chem. A* **2017,** *5* (33), 17240-17247.
